# Integrative Therapies and Pediatric Inflammatory Bowel Disease: The Current Evidence

**DOI:** 10.3390/children1020149

**Published:** 2014-08-25

**Authors:** Sanghamitra M. Misra

**Affiliations:** Academic General Pediatrics, Baylor College of Medicine, 6701 Fannin Street CCC 1540, Houston, TX 77030, USA; E-Mail: smisra@bcm.edu; Tel.:+1-832-723-7680

**Keywords:** inflammatory bowel disease, ulcerative colitis, Crohn’s disease, breastfeeding, diet, PUFAs, probiotics, herbs, helminths, acupuncture, relaxation, exercise

## Abstract

Inflammatory bowel disease (IBD) primarily describes two distinct chronic conditions with unknown etiology, ulcerative colitis (UC) and Crohn’s disease (CD). UC is limited to the colon, while CD may involve any portion of the gastrointestinal tract from mouth to anus. These diseases exhibit a pattern of relapse and remission, and the disease processes are often painful and debilitating. Due to the chronic nature of IBD and the negative side effects of many of the conventional therapies, many patients and their families turn to complementary and alternative medicine (CAM) for symptom relief. This article focuses on the current available evidence behind CAM/integrative therapies for IBD.

## 1. Introduction

Inflammatory bowel disease (IBD) primarily describes two distinct chronic conditions with unknown etiology, ulcerative colitis (UC) and Crohn’s disease (CD). UC is limited to the colon, while CD may involve any portion of the gastrointestinal tract from mouth to anus. These diseases exhibit a pattern of relapse and remission, and the disease processes are often painful and debilitating. The peak incidence of IBD is in patients between the ages of 15 and 25 years. Approximately 25% to 30% of patients with CD and 20% of patients with UC present before the age of 20 years [1]. Unfortunately, children with IBD are generally more likely than adults to present with extensive intestinal involvement and have rapid clinical progression [2,3].

The pathogenesis of CD and UC are not well understood; however, a combination of genetics and environmental factors likely play a role in development of IBD. Genetic predisposition to IBD has been studied extensively. Multiple population studies have shown that relatives of patients with IBD have a significantly higher risk of developing the same condition when compared to the general population [4,5,6]. According to Nunes *et al.* “familial aggregation has been more frequently reported in CD than UC. In first degree relatives, the age-adjusted relative risk of developing the same type of IBD ranges from 2 to 8 for UC and from 5 to 10 in the case of CD [7,8,9,10]”. Twin studies have effectively demonstrated the genetic influence on the development of IBD. In a Swedish twin cohort study of 80 twins with IBD, the concordance rate for monozygotic twins was markedly higher in CD than UC (50% *versus* 19%) and continued to rise over time [11]. The greatest IBD concordance rates have been shown among monozygotic twins, ranging from 20% to 50% in CD and from 14% to 19% in UC twins, whereas in dizygotic twins, concordance rates are as low as 0%–7% in CD and UC twins [12,13]. In an analysis of populations, the prevalence of IBD among the Jewish population is 2 to 4 times higher than in any other ethnic group, being greater in the Ashkenazi than in any other Jewish group [4,9]. 

Numerous theories are emerging on possible environmental factors in the development of IBD, including the use of NSAIDs, the hygiene hypothesis, and early exposure to antibiotics. Although it has not been studied extensively in children, the use of NSAIDs may play a role in the development of IBD. From the prospective cohort study, Nurses’ Health Study I, with data from more than 75,000 women, frequent use of NSAIDs, but not aspirin, seemed to be associated with increased absolute incidence of CD and UC [14]. In those with IBD, NSAIDs have been shown to exacerbate UC and CD symptoms [15]. However, one study in adults concluded that psychological factors contribute to IBD symptom flares and that there was no support for differential rates of the use of NSAIDS, antibiotics or for the occurrence of non-enteric infections related to IBD flares [16]. The hygiene hypothesis proposes that the increasing frequency of immunologic disorders can be attributed to decreased childhood exposure to pathogens and, particularly, enteric pathogens. A few studies have shown that exposure to farm animals early in life may be protective against IBD [17,18]. One study showed a strong protective effect of worm infestations for the occurrence of CD, but not UC [19]. A 2006 study showed that having pet cats before the age of five may be protective against CD [20]. There is emerging concern that early antibiotic use may also alter the immune system and result in autoimmune diseases and IBD. A study by Shaw *et al.* showed that of 36 children with IBD, 21 children (58%) had one or more antibiotic dispensations in their first year of life compared with 39% of controls. Crohn’s disease was diagnosed in 75% of the IBD cases, and those receiving one or more dispensations of antibiotics were at 2.9-times the odds of being an IBD case [21]. The development of IBD is multifactorial, andcontinuing research is needed to better understand the pathophysiology of CD and UC. A more complete understanding of the development of IBD may help create more effective treatments for IBD.

## 2. Conventional Treatment

Allopathic treatment of IBD generally includes corticosteroids to induce remission and 5-aminosalicylic acid (5-ASA) agents, also known as mesalamines, as maintenance therapy. Alternative agents, such as 6-mercaptopurine and azathioprine, are used in more severe disease unresponsive to 5-ASA agents. In patients unresponsive to the above therapies, anti-tumor necrosis factor (TNF) monoclonal antibodies can be effective, but these drugs carry risk of malignancies and severe infections due to their effects on the immune system. Due to the chronic nature of IBD and the negative side effects of many of the conventional therapies, many patients and their families turn to complementary and alternative medicine (CAM) for symptom relief. The incidence of CAM use in pediatric IBD patients from a 2009 multicenter study was 50% [22]. Previous studies documented CAM use in pediatric IBD ranging from 40% to 72% [23,24]. A 2014 survey study of adolescent patients with IBD showed that during the preceding 12 months, 48% regularly used CAM, while 81% reported occasional CAM use [25]. Practitioners caring for pediatric IBD patients should be aware that their patients may be using alternative therapies.

## 3. Integrative Treatment

The integrative treatment of IBD is highly individualized and addresses the “whole” person with a focus on improving quality of life. Integrative practitioners consider a wide array of factors when prescribing a course of treatment. Thorough evaluation of the underlying disorder is the initial step to creating a treatment plan. Assessment of the patient’s diet includes food intolerances and gut permeability, as well as consideration for optimal nutrition and potential nutritional deficiencies. Stool studies, which evaluate the gut flora, specifically the overgrowth of bacteria, are also vital in guiding treatment. Often, understanding which symptoms are most bothersome to the patient helps focus the treatment plan. Herbs and supplements are prescribed if expected to be beneficial and generally considered to be safe. As the field of integrative pediatrics is newly emerging, there are few large studies investigating the safety and efficacy of certain herbs or therapies for particular disease processes. Therefore, practitioners often balance risk and benefit and offer a trial of an herb or therapy that may benefit a patient. In the integrative model, all available interventions are utilized to create a successful plan of care with emphasis on empowering the patient. This article will focus on the current available evidence behind CAM/integrative therapies for IBD.

## 4. Breastfeeding

Many researchers have investigated the relationship of IBD with breastfeeding exposure. Early studies suggested that IBD patients were less likely to have been breastfed as infants. Koletzko *et al.* found that children with CD were more than three times less likely to have been breastfed than their unaffected siblings [26]; however, no association was found between breastfeeding history and the risk of developing UC in childhood [27]. A systematic review examining early-onset IBD (<16 years at diagnosis) concluded that breast milk may be protective, but the quality of existing data is generally poor [28].

## 5. Diet

Diet and nutrition are important aspects of IBD management. As IBD involves the GI tract, patients often search for diets that will alleviate their symptoms. There is no evidence that inflammation of the intestines is directly related to particular foods. However, certain foods may worsen symptoms due to food allergy or food intolerance. Many IBD patients follow a normal diet, but avoid those foods that tend to exacerbate their symptoms. These elimination diets can be helpful, but patients should be cautioned against diets that are so restrictive that they result in nutritional deficiencies. Many IBD patients learn from trial and error which foods they tolerate well and which foods result in GI distress. As food intolerances are usually inconsistent among IBD patients, general dietary recommendations cannot be made for all IBD patients.

Some patients with IBD avoid milk products, because they suffer from lactose intolerance or experience exacerbation of symptoms from milk products. Inadequate calcium intake is present in one-third of IBD patients [29]. Restricting milk products can have negative effects, as it may lead to calcium and vitamin D deficiency [30]. Lactase enzyme supplements can be recommended for better tolerance of milk products.

A common conventional diet option for IBD patients is the low-fiber with low-residue diet. This includes decreasing the intake of raw foods, like fruits, vegetables and nuts, that add bulk residue to stool. This diet may be helpful during flares and for patients with bowel strictures. Families often turn to other diets, like the Maker’s diet, the Specific Carbohydrate Diet^TM^ and the Colitis Five-Step formula. The Maker’s Diet, also known as the Bible diet, focuses on physical, mental, spiritual and emotional health. The diet recommendations include foods that are unprocessed, unrefined and untreated with pesticides or hormones. The Maker’s Diet may be beneficial for patients, but its efficacy has not been studied in children with IBD. The Specific Carbohydrate Diet^TM^ (SCD) is “based on the principle that specifically selected carbohydrates, requiring minimal digestive processes, are well absorbed and leave virtually none to be used for furthering microbial overgrowth in the intestine. As the microbial population decreases due to lack of food, its harmful byproducts also decrease, freeing the intestinal surface of injurious substances. No longer needing protection, the mucus-producing cells stop producing excessive mucus, and carbohydrate digestion is improved. Malabsorption is replaced by absorption” [31]. Evidence shows that the SCD is more effective in CD than in UC. A chart review by Suskind *et al.* suggests that the SCD and other low complex carbohydrate diets may be possible therapeutic options for pediatric Crohn’s disease [32]. A prospective pilot study of the SCD in children with Crohn’s disease showed clinical and mucosal improvements in children with CD using the SCD over 12 and 52 weeks [33]. The Colitis Five-Step formula believes in the elimination of infection with a “natural pathogen killer.” If used with proper diet and physical activity, the formula can put the digestive system back in order. This diet has not been studied in pediatric IBD. The use of any special diet in children with IBD should be coordinated with a knowledgeable practitioner.

Exclusive enteral nutrition (EEN) is the consumption of an elemental or polymeric formula, with the exclusion of all other nutrients, for a period of up to 12 weeks. EEN has become an established and reliable option for the treatment of pediatric IBD [34]. In CD, but not in UC, enteral feeding of defined formula diets as a primary therapy has been shown to induce the remission of active disease. There have been several randomized controlled trials comparing EEN to standard treatment. A study from the Netherlands of 77 Crohn’s disease pediatric patients investigated EEN as either hyperosmolar sip feeds or polymeric formula by a nasogastric tube. In patients completing a six-week course of EEN (*n* = 58), complete remission was achieved in 71%, partial remission in 26% and no response was seen in 3%. The authors concluded that a six-week course of EEN is effective in newly diagnosed pediatric CD, with response rates that seem to be influenced by disease location and nutritional status, but not by type of formula [35]. A study by Knight *et al.* investigated the long-term benefits of EEN in 44 patients. Forty out of 44 patients (90%) responded to enteral nutrition, with a median time to remission of six weeks. Twenty five of these 40 (62%) relapsed, with a median duration of remission of 54.5 weeks (range 4–312). Fifteen (38%) had not relapsed. Twenty one of the 44 (47%) had not received steroids. In those who eventually required steroids, their use was postponed for a median 68 weeks (range 6–190). The authors concluded that EEN can postpone or prevent the need for steroids [36]. A review of five randomized clinical trials in 2000 concluded that “there is no difference in efficacy between enteral nutrition and corticosteroid therapy in the treatment of acute Crohn’s disease in children. Improved growth and development, without the side effects of steroid therapy, make enteral nutrition a better choice for first-line therapy in children with active Crohn’s disease” [37]. A meta-analysis in 2007 also concluded that limited data suggest similar efficacy for EN and corticosteroids [38].

Vitamin supplementation can be beneficial for IBD patients. In general, a daily multivitamin may help improve overall nutritional status. Folic acid deficiency is common in patients taking sulfasalazines. Therefore, all patients on sulfasalazine should take daily folic acid supplementation. Many IBD patients are deficient in vitamin D due to corticosteroid use, malabsorption or lack of sun exposure. IBD patients are also commonly deficient in calcium, sometimes due to avoidance of dairy products. Vitamin D and calcium supplementation are often necessary in children with IBD to ensure optimal growth and healing. Patients with CD and ileal inflammation are often deficient in Vitamin B12 and require supplementation.

## 6. Fish Oil (Polyunsaturated Fatty Acids)

Because of their anti-inflammatory action, omega-3 polyunsaturated fatty acids (PUFAs) may be beneficial in IBD. Of note, the incidence rates of IBD are lower in countries with high fish consumption, like Japan [39]. One study showed that children with IBD have a high risk of omega-6 PUFA depletion, which is related to disease activity [40]. A study by Uchiyama *et al.* in 2010 concluded that an n-3 PUFA food exchange table (N-3DP) significantly increased the erythrocyte membrane n-3/n-6 ratio in IBD patients, and this ratio was significantly higher in the remission group. This suggests that N-3DP alters the fatty acid composition of the cell membrane and influences clinical activity in IBD patients [41]. Although there is evidence that PUFAs can benefit IBD *ex vivo* and in animal models, a systematic review and meta-analyses by Turner *et al.* in 2011 concluded that there are insufficient data to recommend the use of omega-3 fatty acids for the maintenance of remission in CD and UC [42]. Furthermore, a systematic review in 2012 concluded that there is insufficient evidence to recommend omega-3 PUFA in IBD [43].

## 7. Probiotics

Probiotics have been studied extensively for inflammatory conditions, but there are few well-developed studies on probiotic use in pediatric IBD. A study by Shadnoush *et al.* investigated the anti-inflammatory effects of *Bifidobacterium* and *Lactobacillus* in the form of probiotic yogurt. All adult participants (210 IBD patients in remission and 95 healthy controls) were randomized to probiotic yogurt or plain yogurt. After eight weeks of oral yogurt ingestions, serum levels of IL-1β, TNF-α and CRP were significantly decreased in the probiotic yogurt group compared to their baseline values and intervention groups. The serum levels of IL-6 and IL-10 increased significantly after the intervention compared to baseline values and plain yogurt levels (all *p*-values < 0.05). The authors concluded that intestinal homeostasis is a balance between the pro- and anti-inflammatory responses of intestinal immunocytes and could be maintained by probiotics [44]. A small study of 21 adult UC patients by Ishikawa *et al.* looked at *Bifidobacteria*-fermented milk (BFM) for UC and concluded that supplementation with the BFM product of 100 mL per day for one year was successful in maintaining remission and had possible preventive effects on the relapse of ulcerative colitis [45]. *Lactobacillus rhamnosus* GG is a strain of *L. rhamnosus* isolated in 1983 from the intestinal tract of a healthy human which was patented by Sherwood Gorbach and Barry Goldin. A study by Zocco *et al.* investigated the efficacy of *Lactobacillus* GG in maintaining remission in 187 patients with UC. The authors compared *Lactobacillus* GG alone, *Lactobacillus* GG in combination with mesalamine and mesalamine alone. Disease activity index, endoscopic and histological scores were determined at 0, 6 and 12 months and in the case of relapse. The study showed no difference in relapse rate at 6 (*p* = 0.44) and 12 months (*p* = 0.77) among the three treatment groups. Interestingly, treatment with *Lactobacillus* GG seemed to be more effective than standard treatment with mesalazine in prolonging the relapse-free time (*p* < 0.05). *Lactobacillus* GG was found to be effective and safe for maintaining remission in patients with UC, and it may represent a good therapeutic option for preventing relapse [46].

VSL#3 is a potent probiotic composed of eight strains of lactic acid bacteria including, *S. thermophilus* MB455, *B. breve* Y8, *B. longum* Y10 (recently reclassified as *B. lactis*), *B. infantis* Y1, *L. acidophilus* MB443, *L. plantarum* MB452, *L. paracasei* MB451 and *L. bulgaricus* MB453. VSL#3 is available in a single-strength capsule containing 112.5 billion bacteria, a double strength (DS) sachet containing 900 billion bacteria and a JUNIOR packet containing 225 billion bacteria. The dosing of VSL#3 in adults with active UC is 8–16 capsules daily or 1–2 DS sachets daily. For maintenance of remission, the dose is 4–8 capsules daily or one DS sachet. For a child less than two years of age, the dose of VSL#3 for active UC is 1–3 capsules per day and for maintenance is one capsule per day. For those unable to swallow the capsule whole, the capsule may be opened and sprinkled onto a soft food or beverage. In children ages 2–5 years, for active UC, the dose of VSL#3 is 2–3 capsules or one JUNIOR packet per day, and for maintenance, it is 1–2 capsules per day. In children ages 6–11 years of age, the dose of VSL#3 in active UC is 2–4 JUNIOR packets or 4–8 capsules per day, and for maintenance, the dose is 1–2 JUNIOR packets or 2–4 capsules per day. For children ages 12–17 years, the dose of VSL#3 for active UC is 4–8 JUNIOR packets or one DS sachet per day, and for maintenance, it is 2–4 JUNIOR packets or 0.5–1 DS sachets per day. VSL#3 must be kept refrigerated to maintain its potency. VSL#3 is well tolerated. Mild abdominal bloating in the first few days of consuming VSL#3 is the most commonly reported side effect. One randomized, placebo-controlled trial in children showed the efficacy and safety of a highly concentrated mixture of VSL#3 in active UC and demonstrated its role in the maintenance of remission [47]. Similarly, a pilot study of VSL#3 in pediatric patients with mild to moderate UC showed a remission rate of 56% and a combined remission/response rate of 61% [48]. From a meta-analysis of randomized controlled trials (RCTs), administration of VSL#3 can help induce and maintain remission in UC and has been shown to maintain antibiotic-induced remission in relapsing pouchitis. The meta-analysis also concluded that other probiotics have not shown such benefit in UC nor in CD [49]. To date, the efficacy of any probiotic in the treatment of CD has not been clearly demonstrated [50,51]. 

## 8. Herbs

A number of herbs are used by IBD sufferers; however, studies of the benefits and side effects of these herbs are limited. Patients use herbs because of their perceived efficacy, safety and often low cost. Ginger (*Zingiber officinale*) is an herb that is native to Southeast Asia that has been used as a flavoring agent for more than 4,000 years. The root of the ginger plant is most useful for its anti-nausea and anti-bloating effects. Ginger can be consumed directly, as a tea or in capsule form. Caution should be taken in patients on heparin or Coumadin, as ginger may react adversely with these medications. Although ginger has not specifically been studied in pediatric IBD, children with GI symptoms, like nausea, can benefit from ginger [52]. *Boswellia serrata*, an Ayurvedic herb also known as Indian frankincense, is commonly used around the world for IBD. In adults, the *Boswellia* dosage is generally 300–400 mg three-times daily of an extract that contains 37.5% boswellic acids. In a German study of adults with IBD, 36% were taking *Boswellia serrata* extracts [53]. One study by Gupta *et al.* of 30 patients with chronic UC showed *Boswellia* to be similar to sulfasalazine in the UC remission rate. In the study, 14 of the 20 patients treated with *Boswellia* achieved remission, while four of the 10 patients treated with mesalamine achieved remission [54]. A study by Gerhardt *et al.* concluded that therapy with *Boswellia serrata* extract H15 is not inferior to mesalazine in CD [55]. The herb is well tolerated, but one study in 2011 by Holtmeier *et al.* did not find it to be superior to placebo for CD [56]. It should be noted that *Boswellia* can accelerate menstrual flow and may induce miscarriage in pregnant women. Other side effects include nausea, diarrhea and skin rashes. *Boswellia* may also decrease the anti-inflammatory effects of NSAIDs.

Oral *Aloe vera* gel is also used worldwide for IBD, because of its anti-inflammatory properties. *Aloe vera* gel comes from the inner portion of the aloe plant and is a clear, jelly-like substance. *Aloe vera* latex is yellow in color and comes from inside the outer lining of the leaf. *Aloe vera* latex is a potent laxative and should generally be avoided in UC. In active UC, oral *Aloe vera* gel taken for four weeks has been shown to produce a clinical response more often than placebo. This was studied by Langmead *et al.* in a double-blind randomized trial of 44 patients with mild to moderate active UC in which 30 patients were given 100 mL of oral *Aloe vera* gel twice daily and 14 patients were given 100 mL of placebo twice daily [57]. *Aloe vera* gel is generally well tolerated. It should be noted that oral *Aloe vera* may lower blood glucose levels, and there have been a few case reports of acute hepatitis from oral *Aloe vera* [58,59]. Although the mechanism of action of *Aloe vera* is not well understood, there are potential benefits of the herb in IBD.

Curcumin is the principal curcuminoid of the popular South Asian spice, turmeric, which is a member of the ginger family (*Zingiberaceae*). An open label pilot study in 10 adults with UC or CD documented improvement of symptoms with oral intake of 550 mg of curcumin three times a day [60]. Curcumin was shown by Hanai *et al.* to be effective for relapse prevention in adult patients with UC in a randomized, multicenter, double-blind, placebo-controlled trial [61]. A pilot pediatric study of curcumin in 11 patients showed good tolerability of the drug with only one documented side effect, gassiness, in two of the 11 patients. All participants in this pilot study received 500 mg of curcumin twice a day for three weeks, and with use of the forced-dose titration design, doses were increased up to 1 g twice a day at Week 3 for a total of three weeks and then titrated again to 2 g twice a day at Week 6 for three weeks. By using the Pediatric Crohn’s Disease Activity Index (PCDAI) and Pediatric Ulcerative Colitis Activity Index (PUCAI), which are validated measures of disease activity, scores were obtained at Weeks 3, 6 and 9. Three patients saw improvement in their PCDAI/PUCAI score with the use of curcumin [62]. Curcumin is well tolerated and does not cause significant side effects. Reported side effects include stomach upset, nausea, dizziness and diarrhea. Curcumin may inhibit platelet aggregation and increase the risk of bleeding. Therefore, curcumin should be discontinued 1–2 weeks prior to any surgical procedure.

Diet with nutraceutical therapy (DNT) is a specialized therapy that is being used among IBD patients. In an uncontrolled prospective case study of six adolescent patients with CD, all six patients went into remission with discontinuation of all pharmacological drugs within two months of starting DNT. The DNT included adequate caloric and protein intake for catch-up weight gain; elimination of dairy products, certain grains and carrageenan-containing foods; nutraceuticals consisting of fish peptides, bovine colostrum, *Boswellia serrata*, curcumin and a multivitamin daily; the probiotic, *Lactobacillus* GG, twice weekly; and recombinant human GH (rhGH) daily. Three patients remained in sustained remission for four to eight years [63]. The results of this study are impressive. Further research is warranted to evaluate the efficacy of DNT among IBD patients of all ages.

Slippery elm, also known as *Ulmus fulva*, is a supplement made from the powdered bark of the slippery elm tree that is being used in IBD. Although it has not been studied in humans, an *in vitro* study by Langmead *et al.* showed that slippery elm has antioxidant effects. The study also showed that devil’s claw, Mexican yam, tormentil and wei tong ning, a traditional Chinese medicine, have antioxidant properties [64]. A study in adult patients of tormentil extract (TE) showed that it can be beneficial in UC, and the herb appears to be safe in doses up to 3,000 mg per day. Interestingly, an RCT of 40 children in Russia showed that TE can shorten the duration of rotavirus diarrhea and decrease the requirement for rehydration solutions in children. Although this study was performed in children without IBD, the effects of TE may be positive on children with IBD and warrants further study. In a systematic review of 21 RCTs in 2013, for UC, *Aloe vera* gel, *Triticum aestivum* (wheat grass juice), *Andrographis paniculata* extract (HMPL-004) and topical Xilei-san were superior to placebo in inducing remission, and curcumin was superior to placebo in maintaining remission. This study also concluded that in UC, *Boswellia serrata* gum resin and *Plantago ovata* seeds were as effective as mesalamine, whereas *Oenothera biennis* (evening primrose oil) had relapse rates similar to PUFAs. The authors of the systematic review concluded that in CD, *Artemisia absinthium* (wormwood) and *Tripterygium wilfordii* were superior to placebo in inducing remission and in preventing clinical recurrence of postoperative CD, respectively [65]. Blond psyllium, glutamine and wheatgrass are other herbs used by IBD patients. Unfortunately, there is no evidence in the pediatric literature to support their use in IBD.

## 9. Helminthes

It is hypothesized that a lack of exposure to helminthes in the modern era of improved hygiene and healthcare has contributed to the increase of certain autoimmune conditions, like IBD. Ruyssers *et al.* noted: “Although therapy with living helminthes appears to be effective in several immunological diseases, the disadvantages of a treatment based on living parasites are explicit.” [66]. An open trial in adults showed that administration of eggs from the porcine whipworm, *Trichuris suis*, to patients with CD and UC was both safe and resulted in improvement in clinical activity as measured by the Crohn’s Disease Activity Index, Simple Clinical Colitis Activity Index (SCCAI) and the Inflammatory Bowel Disease Quality of Life Index (IBDQ) [67]. To my knowledge, helminth therapy in children with IBD has not been studied.

## 10. Acupuncture

There is a growing body of evidence for the use of acupuncture in adults and children for a variety of diseases. In the realm of IBD, more studies are needed. One prospective, randomized, controlled clinical trial by Joos *et al.* of 29 patients with mild to moderately active UC concluded that both traditional and sham acupuncture seem to offer an added benefit in patients with active UC [68]. A second group also led by Joos *et al.* studied acupuncture in 51 patients with CD via a randomized controlled trial and showed that apart from a marked placebo effect, traditional acupuncture offers an additional therapeutic benefit in patients with mild to moderately active CD [69]. In a systematic review and meta-analysis of 43 RCTs, Ji *et al.* concluded that acupuncture and moxibustion therapy demonstrate better efficacy than oral sulfasalazine in treating IBD. However, “given the limitations of this systematic review and the included literature, definitive conclusions regarding the exact efficacy of acupuncture and moxibustion treatment for IBD cannot be drawn. Extant RCTs still cannot provide sufficient evidence and multicenter, double-blind RCTs with large sample sizes are needed to provide higher-quality evidence.” [70]. Acupuncture may be beneficial for children with IBD to relieve symptoms, such as abdominal pain and nausea, but acupuncture in pediatric IBD has not been formally studied.

## 11. Psychological Health and Relaxation

Stress likely does not cause IBD, but stress can worsen symptoms of IBD for many patients. Using relaxation techniques, like meditation, reflexology, guided imagery and aromatherapy, may help IBD patients feel better. Pediatric patients may also find benefit from reading books and listening to music. A 2010 study examining the use of mind-body therapies in 67 adolescents reported that 62% used prayer, 40% used relaxation and 21% used imagery once/day to once/week for symptom management [71]. A Cochrane Database Review in 2012 concluded that psychological interventions, including psychotherapy, patient education and relaxation techniques, may be beneficial for adolescents with IBD, but the evidence is limited [72]. A study of 39 adults with IBD showed that those who went through a relaxation-training intervention with guided imagery showed statistically significant improvement in anxiety, mood, pain and stress compared to the control group [73]. An interesting study of female adolescents with IBD and their caregivers showed that a one-day intervention (disease-related coping skills, pain management, relaxation techniques, communication and limit setting for the parents) resulted in reduced physical symptoms and improved coping [74]. A study in the Netherlands of adolescents with IBD showed that a psychoeducational intervention can have a positive effect on coping (predictive control, *p* < 0.01), feelings of competence (global self-worth, *p* < 0.05, and physical appearance, *p* < 0.01) and health-related quality of life (body image, *p* < 0.05) [75].

Cognitive behavioral therapy has also been shown to improve global psychosocial functioning and to lessen depressive symptoms in youths with IBD [76]. A recent study by Almadani *et al.* has shown that students with IBD do not adjust to college as well as healthy students. The study used the Short Inflammatory Bowel Disease Questionnaire (SIBDQ) and showed that students with active IBD reported feeling as if they were not doing well for the amount of work they were doing, and students with ulcerative colitis reported irregular class attendance. Almadani *et al.* concluded that “Strategies to increase disease control and provide social and emotional support during college could improve adjustment to college and academic performance, and increase patients’ potential.” [77]. Biofeedback has been shown to improve symptoms of dyssynergic defecation, which is defined as constipation, rectal urgency/incontinence, increased stooling or rectal pain in IBD patients in remission. In a study of 30 adults with dyssynergic defecation who completed biofeedback therapy, 30% had a clinically significant improvement in quality of life as noted by the SIBDQ score and a reduction in healthcare utilization after a six-month period [78]. An interesting survey study of 27 pediatric IBD patients and one of their parents (82% mothers) has shown that parents may be good proxies for quality-of-life ratings for their children, but parents often underreport their child’s health-related quality of life [79]. This suggests that parents should be encouraged to openly discuss their child’s symptoms regularly. Interventions, like counseling, support groups and other available therapies, can then be initiated if needed to help a child cope with the disease. 

Irritable bowel syndrome (IBS) is a disease process distinctly different from IBD. However, there may be overlap of symptoms in many patients. A study in 2002 by Simrén *et al.* investigated quality of life in IBD patients by focusing on 43 adult patients with UC and 40 with CD. All subjects had been in remission for at least one year according to laboratory parameters and clinical and endoscopical appearance. These patients completed four questionnaires evaluating GI symptoms, anxiety, depression and psychological general well-being. Thirty-three percent of UC patients and 57% of CD patients had IBS-like symptoms. The group with IBS-like symptoms (both UC and CD) had higher levels of anxiety and depression and more reduced well-being than those without. The authors concluded that “the prevalence of IBS-like symptoms in IBD patients in long-standing remission is two to three times higher than that in the normal population. Psychological factors seem to be of importance in this process. However, as a group, IBD patients in remission demonstrate psychological well-being comparable to that of the general population.” [80]. There are a variety of integrative treatments available for IBS, like herbs, yoga and acupuncture, but allopathic treatment options for IBS are limited. Tricyclic antidepressants (TCAs) are commonly used to treat IBS. A study by Iskandar *et al.* of 81 IBD patients with residual gastrointestinal (GI) symptoms and 77 IBS patients showed that TCA use led to moderate improvement of residual GI symptoms in IBD patients for whom escalation of IBD therapy was not planned. UC patients demonstrated higher therapeutic success than CD patients [81]. A systematic review of 12 studies investigating antidepressants in IBD concluded that although most studies showed some benefit, there is currently insufficient data to determine the efficacy of antidepressants in IBD [82]. For practitioners, considering the overlap between IBS and IBD may help guide integrative treatment of IBD patients.

## 12. Clinical Hypnotherapy

According to Moser, “Gut-directed hypnotherapy (GHT) has been used successfully in functional gastrointestinal disorders. Few experimental studies and case reports have been published for IBD. GHT increases the health-related quality of life and reduces symptoms. Additionally, GHT seems to have an immune-modulating effect and is able to augment clinical remission in patients with quiescent ulcerative colitis” [83]. Well-controlled studies on clinical hypnotherapy in pediatric IBD are needed.

## 13. Exercise

Exercise can create a sense of well-being. However, exercise can pose challenges for patients with IBD as UC and CD often cause fatigue, abdominal pain, diarrhea and incontinence. All of these symptoms can create barriers to regular exercise. When patients are feeling well enough, they should be encouraged to engage in physical activity appropriate for age and physical fitness level. Low-impact activities, like walking, yoga and tai chi, are good options for most IBD patients. Limited evidence shows that mild to moderate exercise in adults with IBD can benefit the patient without adverse effects [84]. One study showed that lean body mass and physical activity were reduced in 39 children with IBD, whether they were in remission or in the active phase of their disease compared to 39 healthy age-matched controls [85]. As physical activity is necessary for optimal bone and muscle growth, engaging in exercise is important for pediatric IBD patients.

## 14. Smoking

Although it may seem simple, smoking has a complicated effect on IBD. Current smoking increases the risk of developing CD and worsens its course. On the other hand, smoking protects against UC, and in patients with UC, smoking improves its course. Smoking cessation may worsen CD, but improve UC. However, given the harmful long-term effects of smoking on the entire body, smoking cessation should be considered in all pediatric patients [86].

## 15. Conclusions

CAM use in IBD is prevalent and needs to be monitored and researched. As there is potential for improved quality of life and decreased need for allopathic medications with CAM, children and adolescents with IBD will continue to search for treatments that help them with their physical disease and with their ability to cope with their disease. The evidence supporting CAM in pediatrics is small, but growing. General pediatric healthcare professionals and pediatric GI specialists should respond to this trend and address CAM use in their daily practice. This initiative will result in more effective counseling for their families and more integrated care for their patients.
